# Transcranial magnetic stimulation to dorsolateral prefrontal cortex affects conflict-induced behavioural adaptation in a Wisconsin Card Sorting Test analogue

**DOI:** 10.1016/j.neuropsychologia.2016.11.015

**Published:** 2017-01-08

**Authors:** Erica A. Boschin, Rogier B. Mars, Mark J. Buckley

**Affiliations:** a Department of Experimental Psychology, University of Oxford, Oxford, UK; b Donders Institute for Brain, Cognition and Behaviour, Radboud University Nijmegen, The Netherlands and Centre for Functional MRI of the Brain, Nuffield Department of Clinical Neurosciences, John Radcliffe Hospital, University of Oxford, Oxford, UK

**Keywords:** Transcranial magnetic stimulation, Dorsolateral prefrontal cortex, Conflict-monitoring, Behavioural adaptation, Wisconsin Card Sorting Test

## Abstract

A substantial body of literature has proposed a role for dorsolateral prefrontal cortex (dlPFC) in supporting behavioural adaptation during conflict tasks. The vast majority of the evidence in support of this interpretation comes from neuroimaging studies. However, in order to unequivocally ascribe such a role to dlPFC, it is important to determine whether or not it is *essential* for this mechanism, and this can only be achieved by lesioning the area or interfering with its activity. In this study, we investigated the effects of repeated Transcranial Magnetic Stimulation (rTMS) to dlPFC on performance on a conflict version of a Wisconsin Card Sorting Test analogue (used previously in circumscribed lesion studies in monkeys) in neurologically healthy human participants. Our results supported the view of dlPFC as a fundamental structure for optimal conflict-induced behavioural adaptation, as stimulation cancelled out the adaptation effect normally observed on control trials. We show that there is some indication of differential modulation of trial types by stimulation and we hypothesize that this might suggest a role for dlPFC in conflict-induced adaptation that is more specifically concerned with the maintenance of conflict-history information online across trials.

## Highlights

• Dorsolateral prefrontal cortex stimulation abolishes conflict-induced adaptation.
• Adaptation is abolished via differential modulation of trial types.
• Dorsolateral prefrontal areas may aid adaptation via conflict history maintenance.

## 1 Introduction

Dorsolateral prefrontal cortex (dlPFC – defined here as the region occupying Brodmann areas 46, 9/46 and 9 in the superior and middle frontal gyri) is believed to play a fundamental role in exerting top-down control on behaviour. One of the processes dlPFC has been strongly implicated in is the implementation of cognitive control to drive behavioural adaptation during tasks eliciting conflict between two (or more) competing responses. A classic conflict task is the Stroop task (Stroop, 1935), where participants are asked to name the colour a written word is printed in while ignoring the word itself, while conflict is manipulated by using colours congruent (e.g. ‘Red’ in red ink) or incongruent (e.g. ‘Red’ in green ink) with the written word.

In the presence of interference between competing responses (i.e. on high-conflict trials, H), subjects’ performance is negatively affected compared to trials where responses do not interfere with one another (i.e. low-conflict trials, L), with a decrease in speed of response and/or accuracy (e.g. Eriksen and Schultz, 1979; Hedge and Marsh, 1975; Simon and Small, 1969; Simon, 1990; Stroop, 1935; van Veen and Carter, 2005). This is generally defined as a ‘conflict cost’ on performance and is measured as the difference in speed and/or accuracy between H and L trials.

In this context, behavioural adaptation is generally defined as a *reduction* in conflict cost after subjects have been already exposed to conflict on one (or more) immediately preceding trials (also referred to as ‘Gratton effects’ or ‘sequential effects’) (Chen and Melara, 2009, Gratton et al., 1992, Hommel et al., 2004, Nieuwenhuis and Stins, 2006, Ullsperger et al., 2005, Wühr and Ansorge, 2005). Adaptation effects are often attributed to a number of different mechanisms. For example, some accounts point to cognitive control mechanisms becoming engaged on H trials and from there on proactively counteracting the detrimental effects of conflict on subsequent trials by enhancing task-relevant - while suppressing task-irrelevant - information (e.g. Botvinick et al., 2001; Botvinick, 2007; Egner and Hirsch, 2005a). Other accounts emphasize the role of the maintenance of conflict-related information in working memory (Mansouri et al., 2007) in aiding adaptation, or the refreshing/retrieval of task instructions and rules (Badre, 2008, Raye et al., 2002, Roth et al., 2009). While adaptation is likely a result of all these different mechanisms operating in concert with one another, rather than due to one specific mechanism, one important question concerns the localization of these processes within the neural substrate.

Imaging studies have reported high levels of dlPFC activation during adaptation trials in various types of conflict task, such as the Stroop (Egner and Hirsch, 2005a, Egner and Hirsch, 2005b, Kim et al., 2012, Kim et al., 2013), Simon (Kerns, 2006) and flanker (Durston et al., 2003) tasks and suggest a role for this area in supporting behavioural adaptation. While fMRI can provide correlational evidence for the role of a region in a specific cognitive process, neuropsychological studies are essential to determine whether that region is *necessary* for the process. Although neuropsychological evidence on the role of dlPFC in conflict-induced adaptation is currently rather scarce, one study using a conflict analogue of the Wisconsin Card Sorting Test (WCTS) in non-human primates has indeed shown that lesions to dlPFC, impair behavioural adaptation (Mansouri et al., 2007). These findings have also been replicated in human neuropsychological patients using the same task (Boschin et al., in press), and appear consistent with the neuroimaging literature.

Several issues, however, complicate the assessment of neuroimaging findings in neuropsychological patients. One crucial limitation is that, in the vast majority of human clinical cases and unlike the case of laboratory animals that undergo surgical lesions, brain damage is not localized exclusively to the region of interest and might involve, sometimes large, lesions to other brain areas. Furthermore, there is often no opportunity to collect pre-lesion data (which allows to assess the effects of brain damage on a process within-subjects), as well as relatively little control over the length of the period between the lesion and testing and possible compensations that might occur in that interim. One valuable, complementary methodology that can help overcome these limitations is Transcranial Magnetic Stimulation (TMS). TMS allows the experimenter to interfere with neural activity in the brain in a way that has often been described as a temporary ‘virtual lesion’ (Pascual-Leone et al., 1994, Walsh and Cowey, 2000, Walsh and Rushworth, 1999). In this study, we sought to investigate the effects of TMS to dlPFC on measures of conflict-induced behavioural adaptation in the conflict analogue of the WCST.

Several studies have previously used TMS to investigate the mechanisms underlying performance in conflict tasks. However, while areas such as the medial PFC (mPFC) (Hayward et al., 2004, Jin et al., 2010, Neubert et al., 2010, Soutschek et al., 2013, Taylor et al., 2007), pre-motor and motor cortices (Neubert et al., 2010, Praamstra et al., 1999, Stürmer et al., 2000) and posterior parietal cortex (Jin et al., 2010, Stürmer et al., 2007) have been commonly targeted, very few studies have looked at the effects of TMS on dlPFC, especially with regards to adaptation.

TMS to the dlPFC has been found to have no significant effect on conflict cost measures on the current trial (Vanderhasselt et al., 2007, Vanderhasselt et al., 2006, Wagner et al., 2006), but, consistent with the neuroimaging data, it has been found to affect behavioural adaptation on the next trial. Sturmer and colleagues (2007) looked at effects of TMS to dlPFC on adaptation during a Simon task, and found that the reduction in conflict cost usually observed after high-conflict trials was abolished by 20 Hz repetitive TMS (rTMS) applied to the left dlPFC. To the best of our knowledge, this is the only study that has investigated the link between dlPFC and adaptation using TMS.

As previously mentioned, one of the most common explanations for conflict-induced adaptation is that, after cognitive control is engaged, task-relevant information is enhanced, the competing, task-irrelevant information is suppressed and thus the detrimental effect of conflict on performance is reduced (Botvinick et al., 2001, Botvinick, 2007). One would therefore expect that the reduction in conflict costs should involve an improvement in performance on high-conflict trials that were preceded by another high-conflict trial (i.e. HH trials) compared to high-conflict trials that were preceded by a low-conflict trial (i.e. LH trials). However, studies often do not specify whether this is the case (Chen and Melara, 2009; Stürmer et al., 2002; Wühr and Ansorge, 2005). In their investigation into the effects of TMS to dlPFC on adaptation, Sturmer and colleagues (2007) did indeed not specify whether the adaptation effect is abolished through the effects of TMS on specific trial sequences. This is however an important detail, as it can help elucidate what mechanisms are contributing to adaptation in a particular context, for example whether it is via the enhancement of task-relevant behaviour or, as it might be the case when adaptation effects that are entirely due to reductions in speed of response on low-conflict trials following high-conflict trials (i.e. HL trials) (e.g. Horga et al., 2011; Stoffels, 1996), through an increase in caution. Most importantly, as adaptation is likely due to a number of complementary mechanisms, specifying the effects of stimulation on specific trial types can help determine whether a region of interest is important for supporting one mechanism over another, and thus provide a more thorough account of how adaptation might emerge at the network level.

We know from previous work (see “Pilot Study” in the Supplementary Material section) that, in neurologically healthy populations, adaptation effects in the conflict analogue of the Wisconsin Card Sorting Test (WCST) are due to differences in the speed of responses on HH trials compared to LH trials (with faster responses on the former), and that low-conflict trials are unaffected by the nature of the previous trial (i.e. there is no difference in speed of response on HL compared to LL trials). We also know that lesions to dlPFC abolish this effect in both non-human (Mansouri et al., 2007) and human (Boschin et al., in press) primates. In the current study, we followed up on this work by investigating whether TMS to the dlPFC in neurologically healthy participants affects adaptation in the WCST analogue in a manner similar to lesions. Using a paradigm similar to Sturmer and colleagues’ (2007), we applied on-line repetitive rTMS to the left dlPFC during selected trials. Most importantly, given the flexibility afforded by TMS to observe the effects of the ‘virtual lesion’ selectively on a proportion of HH and LH trials while leaving other HH and LH trials unaffected within-subjects, we were able to ask whether the adaptation effect is abolished via increase in speed on LH trials or decrease in speed in HH trials (or both).

We hypothesized that stimulation would abolish the adaptation effect normally observed on non-TMS trials, consistent with findings from lesion studies of dlPFC in monkeys and patients on this task (Boschin et al., in press; Mansouri et al., 2007), and with the human imaging literature suggesting a role for dlPFC in adaptation (Durston, 2003; Egner and Hirsch, 2005a, Egner and Hirsch, 2005b; Kerns, 2006; Kim et al., 2012, 2013). Furthermore, we hypothesized that, if the role of dlPFC in adaptation is to actively engage cognitive control or to enhance task-relevant information while suppressing task-irrelevant information (Botvinick, 2007, Botvinick et al., 2001, Egner and Hirsch, 2005a), HH trials (i.e. the trials that should most benefit from this type of proactive engagement of cognitive control), should be most affected by TMS, with response speed dropping to LH levels, therefore canceling out the adaptation effect. We would not expect LH trials to be affected by disruption to cognitive control mechanisms as a low level of control already characterizes these trials to begin with. On the other hand, if dlPFC is involved in maintaining information about recent conflict-history in working-memory across trials (Mansouri et al., 2007), LH and HH trials should both be affected by TMS, as response speed in both should be dependent on conflict-history. This is because the cognitive system should still be able to implement some degree of cognitive control, but not to efficiently modulate it on a trial-by-trial basis as if it had access to a full history of recent conflict. One hypothesis is that the cognitive system might ‘reset’ to an average level of control that might not be as ‘lax’ as it would normally be after low-conflict trials (thus speeding up LH responses) but not as high as it would normally be after high-conflict trials (thus slowing down HH responses). Alternatively, if dlPFC's role in these kinds of tasks is to refresh/retrieve task instructions and rules (Badre, 2008, Raye et al., 2002, Roth et al., 2009), we predict that LH trials should be most affected, becoming even slower, as these are the trials signaling the need to retrieve task instructions.

## 2 Materials and methods

### 2.1 Participants

32 participants (15 male, mean age 24.18 years) took part in the experiment. Participants were students and staff at the University of Oxford, recruited through advertisement, and received monetary compensation for their participation in the study. All participants had no history of current or previous neurological or psychiatric condition and were not taking any psychoactive medication, as established by a screening questionnaire. All participants were fluent English speakers and had normal or corrected-to-normal vision. They all provided written consent prior to their participation in the study. The research was carried out with the approval of the South Central-Berskshire Research Ethics Service authority and in accordance with the Declaration of Helsinki.

### 2.2 Stimulation sites

In order to investigate whether there might be any subdivisional specificity within dlPFC in aiding adaptation, we targeted two different sites in this region: a more lateral part of dlPFC, which approximately corresponded to areas 46 or 9/46 (from here on referred to as BA 9/46) as well as an area corresponding approximately to midline dorsal area 9 (from here on referred to as BA 9d). Participants were randomly assigned to one of the two experimental groups, BA 9/46 (12 participants) and BA 9d (10 participants), or to a control group (10 participants) that received stimulation at the vertex (see below for details). We targeted left dlPFC as this is an area that has been found to be to be activated during adaptation periods in conflict tasks (eg. Kerns, 2006; Kim et al., 2012; MacDonald, 2000) and, unlike right dlPFC, has been shown to negatively affect adaptation when stimulated (Sturmer et al., 2007).

The site of stimulation in the BA 9/46 group was localized using the Beam F3 Location System (Beam et al., 2009). This system allows the measurement of the location of the F3 electrode position in the 10–20 EEG coordinate system (which has been shown to be a reliable reference point for the localization of dlPFC in the absence of structural brain scans - see, for example Stürmer et al., 2007), by taking into account individual variability in skull sizes.

**Fig. 1:**
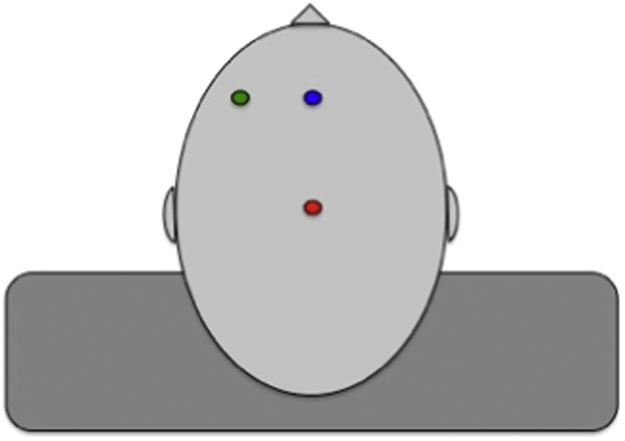
Stimulation sites used in the experiment - BA 9/46 (green), BA 9d (blue) and vertex (red).

BA 9/46 was localized 1 cm caudal to F3, as according to Stürmer and colleagues (2007). The BA 9d site was localized 8 cm rostral to the vertex and 7 cm medial to F3, along the midline, in order to ensure stimulation at a dlPFC site that would be as reliably as possible out of lateral BA 9/46. We chose to target this more superior location within dlPFC as this is an area whose analogous region in the macaque monkey has been shown not to affect performance on the WCST analogue, when lesioned (Buckley et al., 2009, Mansouri et al., 2014), unlike lesions to the more inferior sulcus principalis within dlPFC which is more analogous to human BA 9/46 (Sallet et al., 2013). The control site, the vertex, was localized at a site corresponding to the electrode CZ location in the 10–20 EEG coordinate system, measured as half the distance between inion and nasion and intersecting with half the distance between the two aural canals. The relative location of stimulation sites are illustrated in Fig. 1.

### 2.3 Repetitive Transcranial Magnetic Stimulation

Repetitive Transcranial Magnetic Stimulation was carried out using a biphasic Super Rapid Magstim stimulator (Magstim, Dyfed, UK) and a 70 mm figure-of-eight coil.

First, the Resting Motor Threshold (RMT) was measured individually in each participant. In order to measure the RMT, stimulation was applied to the left primary motor cortex (M1), targeting the site that elicited the largest twitch in the index finger of the participant at the lowest stimulator output (site search started from a spot localized 5 cm laterally and 1 cm rostral of the vertex). The RMT was defined as the intensity needed to produce Motor Evoked Potentials (MEPs) of at least 50 μV in the First Dorsal Interosseus muscle (FDI) of the right hand in at least 5 out of 10 trials. MEPs were recorded using Ag–AgCl electrodes in a belly-tendon montage. The signal was acquired through a CED 1902 amplifier, a CED 1401 analogue-to-digital converter, and the Signal software (Cambridge Electronic Design, Cambridge, UK).

Given the evidence that increases in rTMS frequency lead to longer-lasting effects, as compared to single-pulse TMS (Hallett, 2007), we used slightly lower frequency rTMS than Sturmer (10 Hz) in order to limit the effects of TMS to a shorter, and more precise, time period, while still exploiting the stronger effects of rTMS. As Sturmer and colleagues’ (2007) study indicated that rTMS affects performance when administered in time periods closer to stimulus onset, rather than earlier during the inter-trial period, and that such effect could be larger when stimulation is administered closer to the start of the new trial, we applied TMS at the precise moment of presentation of the test items (see *Task and apparatus* for further details), and therefore at the moment conflict was triggered, requiring rule-retrieval.

Stimulation was performed at 90% of the RMT stimulator output. As a 2.8% reduction in stimulator output for every mm closer to the skull has been recommended (Stokes et al., 2005, Stokes et al., 2007, Stokes et al., 2013), this intensity was within an appropriate range considering the average difference in scalp-cortical surface distance between M1 and the stimulation sites (i.e. approximately 3–4 mm for the Inferior and Middle Frontal Gyri). The stimulation coil was held at a 45° angle off the midline, with the handle pointing in the posterior direction, and rTMS pulse-trains consisted of 3 pulses administered 100 ms apart (10 Hz), on selected high conflict trials.

### 2.4 Task and apparatus

The task used in the study was a computerized analogue of the Wisconsin Card Sorting Test, with trial-by-trial modulation of conflict levels, which was originally developed by Mansouri et al. (2007). The task was programmed using Turbo Pascal (Borland), run in DOS on a desktop PC and presented on a 20.1” colour touchscreen (TFT LCD TS200H GNR), which was used to collect responses. Participants sat at a distance of 40 cm from the screen, resting their chins on a chin-rest, in order to stabilize their head position throughout the experiment. They were instructed to respond using the index finger of their dominant hand to touch the items on the screen.

**Fig. 2:**
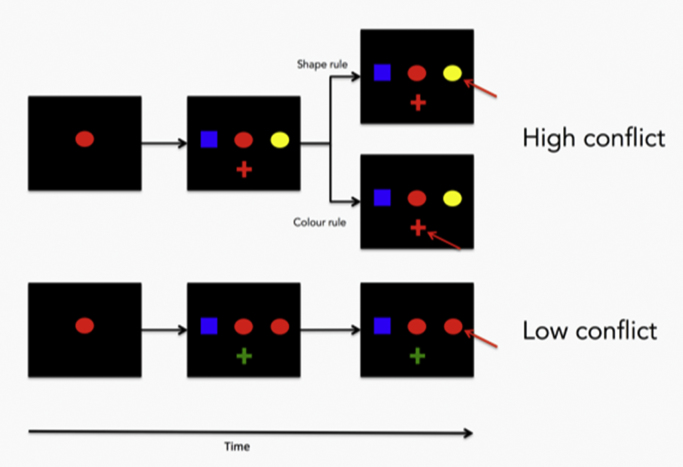
The conflict Wisconsin Card Sorting Test analogue **-** An example of a typical trial in the WCST analogue in the high-conflict condition (top) or the low-conflict condition (bottom). The correct choice is indicated by a red arrow.

The stimulus set consisted of all combinations of six possible shapes (triangle, circle, square, hexagon, ellipse or cross), each 2.4° of visual angle in width and 2.4° in height, in six possible colours (red, green, blue, cyan, magenta or yellow), for a total of 36 possible stimuli, and were presented against a black background. The sample item was always presented in the centre of the screen, and the test items were presented 2.6 degrees to the right, left and bottom of the sample item (Fig. 2).

A typical trial was structured as follows. At the start of the trial, a random sample item was presented in the centre of the screen. Participants were instructed to touch the sample item when they were ready to start the trial. Once the sample item was touched, three test items appeared on the screen. Participants were instructed to carry out a matching-to-sample task, where the rule for matching could be either “match by shape” (i.e. pick the test item that shared the same shape as the sample item) or “match by colour” (i.e. pick the test item that shared the same colour as the sample item). All items remained on the screen until a response was made or until 10 s had elapsed. Correct trials were identified by a high-pitch sound and the correct item remaining on the screen (while the incorrect, unselected items disappeared), indicating positive feedback. Incorrect trials were identified by a low-pitched sound accompanied by the presentation of a large, gray circle, indicating negative feedback. Two seconds after the response, another sample item appeared on the screen, indicating the start of another trial.

Conflict levels were manipulated by changing the degree of feature overlap between the sample and test items. In low-conflict L trials, one of the test items was identical to the sample item (i.e. matched the sample item on both the relevant – for example colour – and irrelevant – for example, shape – dimension), while the other two test items shared neither shape nor colour with the sample item. In high-conflict (H) trials, one of the test items matched the sample item only on the relevant dimension (e.g. colour), while another matched the sample item only on the irrelevant dimension (e.g. shape). A third test item shared neither colour nor shape with the sample item. H and L trials were presented in a randomized order throughout the session, irrespective of the currently reinforced rule (examples of a H and a L trial are presented in Fig. 2).

Participants were informed that one rule would be ‘correct’ for several trials and then the other would be ‘correct’ for several trials, with the rules switching unpredictably during the task, such that they would have to periodically reassess which rule was currently relevant in order to perform the correct response. The rule switch occurred only once an accuracy criterion of 85% on the current rule had been reached over the preceding twenty trials. Participants carried out a total of 4 blocks, with 150 trials in each block.

Since the equivalent of low-conflict trials in another version of a WCST analogue have been shown not to be affected by TMS (Ko et al., 2008), and in order to maximize the number of pairs of trials that could be reliably considered free of TMS effects carrying over from previous trials, only H trials were stimulated. rTMS pulse-trains were applied to half the total number of H trials. On TMS trials, pulse-trains were triggered at the time the sample item was touched by the participant, thereby initiating the trial. Testing sessions were pre-programmed so that participants would not be able to anticipate stimulation, and in such a way that an equal number of LH and HH trials were available for stimulation in each session.

As rTMS effects can outlast the period of stimulation itself (Hallett, 2007) and we were interested in the effect of stimulation on adaptation (and therefore the interaction between the nature of the previous trial (L or H) and stimulation on the current trial) we ensured that possible additive effects of a TMS trial on subsequent trial were controlled for. In order to allow for the effects of stimulation to dissipate, pulse-trains were never administered less than 3 trials apart. Trials were considered “clear” (i.e. non-TMS) trials only if the previous two trials were both also non-TMS trials. Similarly, TMS was administered only on trials that were preceded by two clear trials. A block contained, on average, 25 TMS trials and 25 clear trials. Response times and errors were recorded for analysis.

## 3 Results

In order to reduce the impact of occasional high response time values without arbitrarily removing outliers from the analysis, and, more importantly, to maintain consistency in the type of measure used for analysis between this and other studies that used the WCST conflict analogue (Boschin et al., in press; Mansouri et al., 2015, Mansouri et al., 2007), all analyses were carried out on the speed of target selection (STS). This measure was computed by taking the reciprocal of the response time data, so that low values correspond to slow responses and high values correspond to fast responses. STS values were calculated individually for each trial, and all averages and differential values considered in the analyses were obtained from these individual STS values. The overall number of errors was too low to carry out any meaningful analysis on error data (on average, the number of errors that were not due to rule-switching accounted for only 0.3% of all trials), therefore the analysis was carried out on STS data only.

In order to verify the presence of conflict and adaptation effects in our sample population, we carried out two *t*-tests to explore the difference in STS values between low- and high-conflict trials, as well as between LH and HH trials. There was a significant difference in STS values between low- and high-conflict trials (t_(31)_ =11.69, p<0.001), indicating the presence of a conflict effect, with larger STS values for low-conflict (mean =1.43, sd =0.15) than high-conflict (mean =1.31, sd =0.16) trials. In other words, as expected, participants were faster in responding to low-conflict trials than high-conflict trials. There was also a significant difference in STS values between LH and HH trials (t_(31)_=4.99, p<0.001), with larger STS values for HH (mean=1.34, sd=0.17) than LH (mean=1.29, sd =0.17) trials. This confirms the presence of an adaptation effect, with faster responses to HH than LH trials in the overall population. Descriptive statistics for these results, as well as the following group-based results are also reported in Table 1.

At an individual level, a small number of participants showed a negative adaptation effect on non-TMS trials (i.e. faster responses on LH compared to HH trials). As we were interested in investigating the effects of TMS on a standard (i.e. positive) adaptation effect, these participants were excluded from the analysis. After these exclusions, 9 participants were entered in the BA 9/46 group analysis, 7 participants in the BA 9d group analysis and 9 participants in the control group analysis.

A 2x2×3 mixed ANOVA was carried out on the STS data, with Adaptation (LH or HH) and Stimulation (TMS or non-TMS) as the within-subject factors and Group (BA 9/46, BA 9d and Vertex) as the between-subject factor. The ANOVA indicated a main effect of adaptation (F_(1,22)_=24.22, p<0.001), with lower STS values for LH trials (mean=1.31, sd=0.18) than HH trials (mean =1.35, sd =0.18). This once again confirms the presence of an adaptation effect, with faster responses to HH trials than to LH trials. There was a significant main effect of Stimulation (F_(1,22)_=6.46, p=0.019), with higher STS values for TMS trials (mean=1.35, sd=0.19) than non-TMS trials (mean=1.31, sd=0.18), suggesting that stimulation sped up responses overall, as well as a significant interaction between Adaptation and Stimulation (F_(1,22)_=21.85, p<0.001). The adaptation effect (measured as the difference in STS between HH and LH trials) was smaller on TMS trials (mean =0.01, sd =0.05) than on non-TMS trials (mean =0.07, sd =0.05). This indicates that stimulation abolishes the adaptation effect observed on control trials. Importantly, while there was no significant main effect of Group (F_(1,22)_=1.52, p<0.05), nor interaction between Group and Stimulation (F_(1,22)_=1.39, p<0.05), the three-way interaction between Adaptation, Stimulation and Group was significant (F_(2,22)_=3.53, p=0.047), indicating that the modulation of adaptation by TMS differs amongst the three groups. In order to investigate the significant three-way interaction, we carried out three separate 2×2 repeated-measures ANOVA for the three groups.

**Fig. 3:**
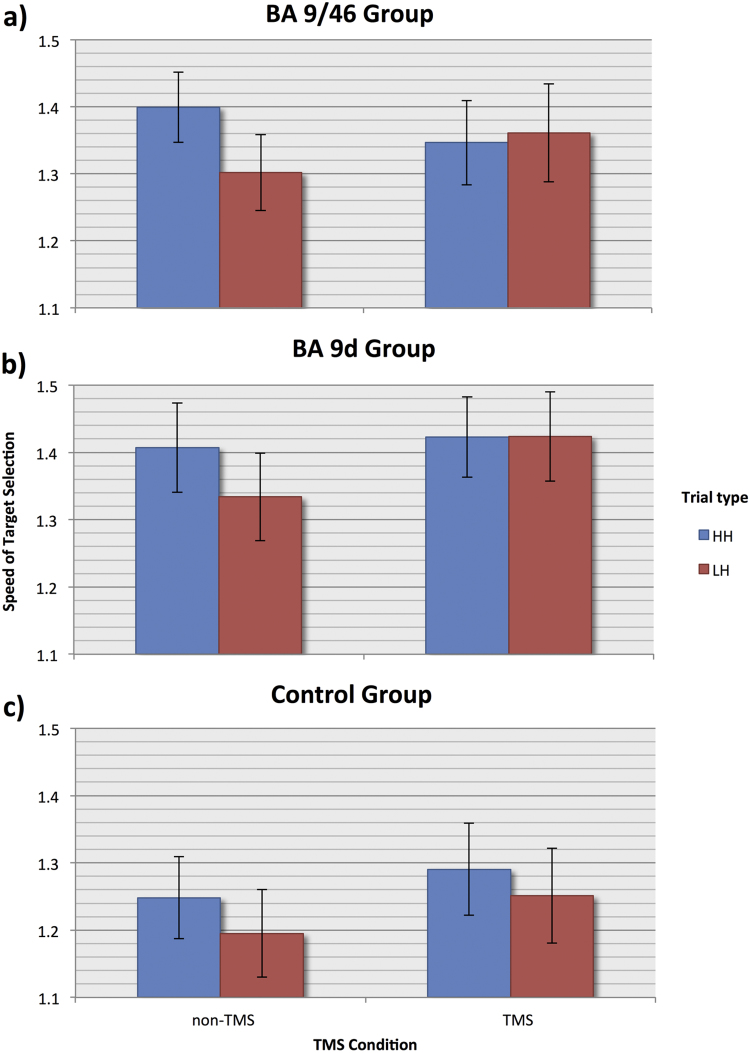
Effects of TMS on Speed of Target Selection (STS) to HH and LH trials – Higher STS values for HH than LH trials indicate an adaptation effect. (a, b) BA9/46 and BA9d group. Adaptation is only present on non-TMS trials (leftmost columns), and is abolished by stimulation (rightmost columns). (c) Control group (vertex). Adaptation is present on both non-TMS (leftmost columns) and TMS trials (rightmost columns).

In the BA 9/46 group, there was a significant main effect of Adaptation (F_(1,8)_=7.84, p=0.023), with lower STS values for LH trials (mean=1.33, sd =0.19) than HH trials (mean=1.37, sd=0.16), confirming the presence of an adaptation effect for this group. The main effect of Stimulation was not significant (F_(1,8)_<1), but there was a significant interaction between Adaptation and Stimulation (F_(1,8)_ =14.56, p=0.005). Once again, we observed a smaller adaptation effect on TMS trials (mean =−0.01, sd =0.05) than on non-TMS trials (mean =0.10, sd =0.07), indicating that the difference in speed between HH and LH trials that is present on control trials is abolished by stimulation. We investigated this interaction further by carrying out Bonferroni-corrected paired-samples *t*-tests on the speed of response to HH and LH trials under the two Stimulation conditions, in order to determine whether the adaptation effect is abolished by the selective effect of stimulation on one specific trial type. While, numerically, TMS LH trials were faster than non-TMS LH trials (mean=1.36, sd =0.22), this difference was not significant (t_(8)_ =−1.87, p>0.05). Likewise, while there was a numerical difference between TMS (mean=1.35, sd =0.19) and non-TMS (mean =1.40, sd =0.16) HH trials, this difference was not significant (t_(8)_=1.51, p>0.05). Stimulation of BA 9/46, therefore, appears to affect the overall adaptation effect, with no selective effect on either type of trial (see Fig. 3a).

In the BA 9d group, STS values for HH trials were numerically higher (mean =1.41, sd =0.16) than LH trials (mean =1.38, sd =0.17), but the main effect of Adaptation narrowly failed to reach significance (F_(1,6)_ =5.36, p=0.060). The main effect of Stimulation was significant for this group (F_(1,6)_ =13.23, p=0.011), with higher STS values on TMS trials (mean =1.42, sd =0.16) than non-TMS trials (mean =1.37, sd =0.17), indicating that stimulation sped up responses overall. The interaction between Adaptation and Stimulation was significant (F_(1,6)_ =8.47, p=0.027), indicating a modulatory effect of TMS on adaptation. As in the BA 9/46 group, stimulation abolished the adaptation effect (mean =−0.001, sd =0.07) observed on non-TMS trials (mean =0.07, sd =0.02). We once again further investigated this interaction with two Bonferroni-corrected paired-samples *t*-tests, which revealed a significant difference (t_(6)_ =−6.09, p=0.001) in the speed of response between TMS LH trials and non-TMS LH trials, with faster responses to the former (mean =1.42, sd =0.18) than the latter (mean =1.33, sd =0.17). There was no significant difference between TMS and non-TMS HH trials (t_(6)_ <1). This indicates that stimulation of BA 9d specifically modulated the speed of response on LH trials (Fig. 3b).

Lastly, the control group (Fig. 3c) showed a significant main effect of Adaptation (F_(1,8)_ =13.43, p=0.006), with higher STS values for HH (mean =1.26, sd =0.18) than LH trials (mean =1.22, sd =0.20), indicating a typical adaptation effect. There was also a significant main effect of Stimulation (F_(1,8)_ =6.23, p=0.037), with higher STS values for TMS (mean =1.27, sd =0.20) than non-TMS trials (mean =1.22, sd =0.18), indicating that stimulation sped up responses overall. Unlike the other two groups, however, the control group showed no significant interaction between Stimulation and Adaptation (F_(1,8)_ =1.07, p=0.331). This suggests that stimulation of the control region, the vertex, does not affect adaptation in the conflict analogue of the WCST.

In order to further investigate subdivisional differentiation within dlPFC, we ran a further 2x2×2 mixed ANOVA directly comparing only the BA 46/9 and BA 9d groups. The main effect of Adaptation (F_(1,14)_ =12.76, p=0.003) was significant, as well as the interaction between Stimulation and Adaptation (F_(1,14)_ =21.40, p<0.001), confirming once more that, in both groups, TMS stimulation affected the adaptation effects. However, the three-way interaction between Stimulation, Adaptation and Group was not significant (F_(1,14)_<1), indicating that TMS did not differentially affect adaptation effects depending on stimulation site.

## 4 Discussion

In this study, we investigated the role of the dlPFC in conflict-induced behavioural adaptation using TMS. We were interested in whether applying TMS to the left dlPFC during high conflict trials of the WCST analogue would affect performance to the same extent as lesions to this area do, i.e. by abolishing the adaptation effect normally observed in this task. Furthermore, we wished to investigate whether any the effect would be selective to specific trial types (i.e. the speed of response to HH as opposed to LH trials) or only be observable as a reduction of the overall adaptation effect (i.e. the difference between LH and HH trials).

We found that TMS to both dlPFC areas (BA 9/46 and BA 9d) cancelled out the adaptation effect, reducing the response speed difference that is normally observed between LH and HH trials. This finding is consistent with previous work showing that dlPFC is an essential structure to support adaptation in the WCST analogue in both human patients and non-human primates (Boschin et al., in press; Mansouri et al., 2007). They are also consistent with the body of literature showing that conflict-induced behavioural adaptation is negatively affected by TMS to structures thought to be involved in conflict-related performance (Hayward et al., 2004, Jin et al., 2010, Neubert et al., 2010, Praamstra et al., 1999, Soutschek et al., 2013, Taylor et al., 2007) and with TMS and imaging literature indicating that dlPFC in particular is involved in adaptation (Durston et al., 2003, Egner and Hirsch, 2005a, Egner and Hirsch, 2005b, Kerns, 2006, Kim et al., 2012, Kim et al., 2013, Stürmer et al., 2007). Although some studies (Ko et al., 2008, Wagner et al., 2006) reported no effect of left dlPFC stimulation on WCST paradigms, our results are not necessarily inconsistent with these findings, as we specifically looked at whether TMS affected the way the nature of previous trials modulated performance on the current trial in a conflict version of the task, rather than overall performance on the standard version of the WCST.

As far as the selective effect of TMS on specific trial types (HH or LH) is concerned, although both trial types were affected by stimulation, our data suggested some differentiation in the effects of TMS to different sites within dlPFC. While TMS to BA 9/46 appeared to cancel out the adaptation effect via unspecific modulation of trials trial types (LH trials were numerically, but not significantly, faster, and HH trials were numerically, but not significantly, slower, when TMS was applied), TMS to BA 9d appeared to cancel out the adaptation effect specifically through an increase in speed on LH trials. However, this differentiation was not robust because when the BA 46/9 and BA 9d groups were directly compared (without control group) the analyses indicated that LH trial modulation was not significantly different between the two groups.

These findings may have potential implications for the different hypotheses regarding the mechanisms by which dlPFC may implement adaptation. The absence of an adaptation effect, and a slower speed of response on HH trials following stimulation is consistent with the hypothesis that dlPFC might be involved in engaging cognitive resources in order to minimize the effects of conflict on subsequent trials, either through the enhancement of task-relevant information, sustained attention or rule-maintenance (Botvinick et al., 2001, Botvinick, 2007, Egner and Hirsch, 2005a). However, faster responses on LH trials are not consistent with this same hypothesis. Being the first trial after a change in the level of conflict, LH trials represent a context where cognitive control is a necessary requirement for accurate and fast responses. Equally, faster responses on LH trials are also inconsistent with a role of dlPFC in the reactivation or retrieval of recent representations (Badre, 2008, Raye et al., 2002, Roth et al., 2009), as interference with these mechanisms should result in slower responses in this type of trials.

Abolition of the adaptation effect that is dependent on modulation of both HH and LH trials may be consistent with a role of dlPFC in maintaining conflict-related information, as maintenance of conflict information might not refer strictly to the maintenance of information regarding high levels of conflict. Cells in dlPFC have been found to respond to both low- and high-conflict trials of the WCST analogue, as well as being sensitive to both LH and HH trials during the inter-trial period (Mansouri et al., 2007). Furthermore, dlPFC activity on the current high-conflict trial has been found to increase as the number of preceding consecutive low-conflict trials increased and to decrease as the number of preceding consecutive high-conflict trials increased, a pattern that was also reflected in the subjects’ response times (Durston et al., 2003). This suggests that dlPFC is not simply recruited in the presence of sustained high conflict (i.e. when several H trials occur in a row), but rather during a narrow time-window centered on recent changes in conflict levels from low to high conflict, and particularly when the recent conflict history was stable (i.e. an H trial occurring after several L trials in a row). dlPFC may therefore be maintaining recent conflict-history “online”, and be particularly sensitive to changes in this type of contextual information. Consequently, loss of this information, following dlPFC damage or stimulation, might affect the cognitive system's ability to take into account the nature of recent trials in order to regulate behaviour on the current trial, whether this is in terms of sustaining performance across trials (HH trials) or responding to changes with respect to a previous trial (LH trials).

We hypothesize that this pattern of results might be explained by a conflict-history based modulation of rule value.

As the WCST is a task that involves periodical rule-switching across blocks, each block should strongly bias one rule over the other, with this bias being reflected in the value assigned to either rule. In other words, in a specific block, on average, the value of the relevant rule should be higher than the value of the irrelevant rule. These values might however also be further modulated within blocks according to trial-by-trial demands. On low-conflict trials, when either rule can be applied for a correct response, the value of the currently relevant rule might decay slightly, while the value of the irrelevant rule might increase slightly, leading to slower responses on subsequent high-conflict trials (LH). Conversely, on high-conflict trials, when the two rules compete with each other but only one can be applied for a correct response, the value of the currently relevant rule might increase slightly, while the one for the irrelevant rule might decay slightly, leading to faster responses on subsequent high-conflict trials (HH). Such trial-by-trial fluctuations in value would therefore occur within a window centered on the average values for each rule within a block.

However, should the information regarding recent trial history be lost (as it might be the case following dlPFC damage or stimulation), the cognitive system might reset to the average values for each rule within a particular block. Therefore, on average, LH trials that would normally be characterized by a lower than average value for the relevant rule, will ‘reset’ to the higher average value, leading to faster responses. On the other hand, HH trials that, on average, would normally be characterized by a higher than average value for the relevant rule, will reset to the lower average value, leading to slower responses.

One possible pathway for this trial-by-trial modulation of rule value might a network including dlPFC and orbitofrontal cortex (OFC). There are strong reciprocal connection between dlPFC and OFC (Petrides and Pandya, 1999, Yeterian et al., 2012), and the latter has been shown to be crucially involved in maintaining and updating the value of the relevant rule in the standard WCST analogue (Buckley et al., 2009), as well as in selecting the appropriate rule in the conflict analogue of this task (Mansouri et al., 2014), in the monkey. Following loss of dlPFC contributions, the network might have to rely more heavily on OFC for performance. This should lead to a loss of trial-by-trial adaptation, measured as differences in speed of response, but maintenance of accuracy levels, since OFC should still hold a higher value for the currently relevant rule than the irrelevant rule. Indeed, in the monkey, lesions to dlPFC, while abolishing speed-related adaptation effects, have been shown not to cause impairments in overall accuracy on the conflict analogue of the WCST, compared to controls (Mansouri et al., 2007). Loss of OFC contributions, on the other hand, should cause both the obliteration of speed-related adaptation effects as well as impairment in accuracy, due to the inability to correctly modulate rule values and to select the relevant rule. Mansouri et al. (2014) did indeed report this pattern of results in monkeys with OFC lesions, with loss of adaptation effects and a decrease in correct responses on high-conflict trials, regardless of the nature of the previous trial. This body of evidence is therefore consistent with a system whereby recent conflict history, encoded in dlPFC, is used to modulate OFC's contributions in the selection and updating of behavioural rules.

Although our results strongly corroborate the hypothesis that dlPFC is a fundamental structure for conflict-induced adaptation, and that adaptation is impaired by damage or interference with dlPFC activity, we feel that our findings regarding the mechanisms that might be at play during specific trial types and their modulation are still very tentative at this stage, and further research is required to truly elucidate these processes. Future research aiming to investigate the effects of TMS or lesions (as well as the functional interactions between dlPFC and other areas) on sequences of trials (e.g. LLLH, LLHH, LHHH, etc.), or to manipulate the volatility of conflict levels (i.e. how often they change within a session) or the frequency of high- versus low-conflict trials during the task could shed more light on the specific dynamics involved in trial-by-trial modulation of conflict related behaviour, not only in the WCST, but also in other types of conflict tasks.

As far as any potential subdivisional specificity within dlPFC is concerned, more precise stimulation of the regions of interest, aided by structural scans, could help clarify these findings. As we could not obtain a scan of each of our subjects’ brain, we relied on scalp landmark-based measurements for the localization of our stimulation sites. While we based our measurements on previous literature demonstrating their reliability (Stürmer et al., 2007), this nonetheless led to some limitations for the level of precision required to investigate subdivisional specificity. For example, in order to ensure that our BA 9d group was stimulated reliably outside of BA 9/46, we applied the coil along the midline. This approach most likely resulted in bilateral stimulation and, as right dlPFC stimulation has been previously shown not to affect adaptation, albeit in a different task (Stürmer et al., 2007), it might have impacted the final results. There are also inherent limitations in the use of rTMS due to the spread of stimulation effects from the stimulation site to interconnected areas (Bestmann et al., 2008, Bestmann et al., 2004), which likely affected the strongly interconnected BA 46/9 and BA 9d in our experimental paradigm, making it difficult to clearly discern any conclusive differentiation. This issue also poses some limitations in the comparison of our findings with monkey data. While previous evidence showed that, in the monkey, lesions to an area analogue to BA 9d in the human brain does not affect performance on the WCST analogue or its conflict version (Buckley et al., 2009, Mansouri et al., 2014), TMS to BA 9d did affect adaptation in our sample. However, given the aforementioned limitations, we feel the data is too preliminary to describe this as a robust inter-species difference.

To summarize, we showed that stimulation to left dlPFC regions abolishes the conflict-induced adaptation effect normally observed in a conflict analogue of the WCST. This is consistent with data from neuroimaging, neurophysiology, and neuropsychology (both in non-human primates and in humans tested on the same task). Such convergence of correlational (fMRI, neuronal recording) and intervention (lesions, TMS) findings, particularly across species, add weight to the conclusion that dlPFC plays a fundamental role in driving adaptation to conflict. We found some evidence of selective modulation of specific trial types that might underpin the specific mechanisms by which dlPFC supports adaptation, as well as some suggestion that there might be potential subdivisional specificity within dlPFC, which could benefit from further investigation. We hypothesize that dlPFC might be involved in maintaining conflict history information online across trials, which is then used to modulate behavioural strategies in response to contextual changes.

## Author contributions

EAB and MJB designed and conducted the study, performed the analyses, and wrote the paper; RBM trained the first author in the use of TMS and advised during manuscript preparation.

## Figures and Tables

**Table 1:** Descriptive Statistics – Means and standard deviations for all subjects (n. 32) and each experimental group (excluding subjects that showed a null or negative adaptation effect, n. 25), for each conflict level and TMS condition.

**Group**	**TMS**	**Low**		**High**		**HH**		**LH**	
		*Mean*	*SD*	*Mean*	*SD*	*Mean*	*SD*	*Mean*	*SD*
All	On			1.35	0.19	1.36	0.17	1.34	0.20
	Off	1.43	0.15	1.31	0.16	1.34	0.17	1.29	0.17

Left 46	On			1.35	0.21	1.35	0.19	1.36	0.22
	Off	1.47	0.14	1.33	0.16	1.40	0.16	1.30	0.17

Midline 9	On			1.43	0.16	1.42	0.16	1.42	0.18
	Off	1.46	0.16	1.35	0.16	1.41	0.17	1.33	0.17

Vertex	On			1.27	0.20	1.29	0.20	1.25	0.21
	Off	1.34	0.15	1.21	0.18	1.25	0.18	1.19	0.19
